# Metallothionein 2 and Heat Shock Protein 72 Protect *Allolobophora chlorotica* from Cadmium But Not Nickel or Copper Exposure: Body Malformation and Coelomocyte Functioning

**DOI:** 10.1007/s00244-016-0276-6

**Published:** 2016-04-02

**Authors:** Joanna Homa, Stephen R. Stürzenbaum, Elzbieta Kolaczkowska

**Affiliations:** Department of Evolutionary Immunology, Institute of Zoology, Jagiellonian University, Gronostajowa 9, 30-387 Krakow, Poland; Analytical and Environmental Sciences Division, Faculty of Life Sciences & Medicine, King’s College London, London, UK

## Abstract

Earthworms serve as good indicators of heavy metal contamination due to their innate sensitivity towards soil pollution. However, to date, not many studies have focused on endogeic earthworms, such as the omnipresent *Allolobophora chlorotica*. The current study was designed to verify whether this earthworm could serve as a novel distinctively susceptible species for environmental contamination studies. We show that the dermal exposure to Cu, Ni, and Cd affected the mortality and morphology of *A. chlorotica*, and the number and functioning of coelomocytes. These features particularly were pronounced in animals treated with Ni and Cu and interestingly to a lesser extend with Cd. In contrast, Cd induced a strong expression of metallothioneins (MT-2) and heat shock proteins (HSP72). The presence of MT-2 was detected not only in coelomocytes but also in the intestine, blood vessels, and epidermis. In conclusion, *Allolobophora chlorotica* coelomocytes are adopted to respond differentially to various heavy metals, generating powerful response towards potentially most dangerous exogenous non-essential elements.

Earthworms are one of the most important macroinvertebrates, because their presence influences soil properties and availability of resources to their inhabitants (Kooch and Jalilvand 2008). Likewise, when the well-being of earthworms is impaired, for example due to soil contamination, with pesticides or heavy metals, important soil functions can be compromised (Calisi et al. 2014; Giska et al. 2014; Leitão et al. 2014). For this reason, earthworms are environmental sentinels and biological indicators of soil quality and pollution. The earthworm coelomic cavity contains coelomocytes, immunocompetent cells classified as amoebocytes, and eleocytes/chloragocytes (Engelmann et al. 2004; Kurek et al. 2007). Whilst amoebocytes can recognize foreign materials (e.g., pathogens) and are involved in phagocytosis and encapsulation (Cossarizza et al. 1996; Engelmann et al. 2004; Kalaç et al. 2002), eleocytes store endogenous materials such as glycogen and lipids (Roots and Johnston 1966), as well as exogenous compounds, such as pigments or metals (Prentø 1979, Fisher and Molnár 1992). Chloragocytes also have been associated with the immune defense, because they secrete bacteriostatic compounds (Valembois et al. 1992) and are involved in encapsulation and in the formation of brown bodies (Cooper and Stein 1981; Field et al. 2004; Valembois et al. 1992, 1994). Moreover, all coelomocytes are involved in heavy metal trafficking within the earthworm body (Homa et al. 2007; Honeycutt et al. 1995).

An exposure to heavy metals results in the upregulation of numerous genes resulting in the expression of proteins involved in the detoxification of metals and/or repair of lesions. In particular, the expression of metallothioneins (MTs) and heat shock proteins (HSPs) increase in the presence of heavy metals (Calisi et al. 2009; Homa et al. 2005; Kammenga et al. 2000). Indeed, metallothioneins play a crucial role in the metabolism, transport, homeostasis, and detoxification of essential and nonessential metals (Calisi et al. 2009, 2014; Dallinger 1996; Roesijadi 1996). There are two MT isoforms, M1 participates more in physiological activities, whereas MT2 binds nonessential metals, such as cadmium (Morgan et al. 2004).

Heavy metals also can induce the expression of cytoprotective heat shock proteins (HSPs) functioning as molecular chaperons controlling protein–protein interactions and preventing redundant protein aggregation (Kiang and Tsokos 1998; Luan et al. 2010; Moseley 2000; Nadeau et al. 2001; Piano et al. 2004). In particular, the family of 70-kDa heat shock proteins (HSP-70) represents one of the most abundant stress proteins, and among them Hsp72 protects cells against cellular stress (cytoprotection), also in earthworms (Nadeau et al. 2001). Any stressor, including heavy metals, affects the immune system equilibrium disturbing not only the production of immune-related proteins (Nadeau et al. 2001) or the cells cycle (Shackelford et al. 1999) but also more general processes, such as worm behavior, reproduction, offspring viability, ageing, and population level effects (Homa et al. 2003; Liess 2002). Whilst the epigeic *Eisenia fetida* is widely used in acute toxicity tests (Maboeta et al. 2004), the endogeic *Allobophora chlorotica* is less well studied. However, unlike *E. fetida*, which is restricted to territories rich in decaying organic material (compost), *A. chlorotica* can be found in a wide range of habitats, including forests, meadows, agriculture areas, and city gardens (Sizmur et al. 2011), and therefore feeds on and thus stays in constant contact with soil. This renders *A. chlorotica* an excellent candidate for comparative ecotoxicological studies on different ecosystems. Indeed, *A. chlorotica* are sensitive to heavy metal (Zn > Pb > Cd > Cu) polluted soil (Homa et al. 2003), and their coelomic fluid contain an abundance of amoebocytes and eleocytes (Kurek et al. 2007).

The goal of the current study was to establish whether endogeic earthworm species, such as *A. chlorotica*, are suitable biomonitors of metal contamination. Additionally, we aimed to identify immunological parameters that would be modulated differentially by various distinct heavy metals. Therefore, the current study challenged *A. chlorotica* to a range of cadmium, copper, and nickel doses. Together with Hg and Pb, Cd is one of the big three heavy metal poisons and is not known for any essential biological function, whereas Ni and Cu are among heavy metals essential for healthy plant and animal growth (Wuana and Okieimen 2011). The mortality rate and malformation of the worm body were monitored, changes in eleocyte composition, lysosomal membrane stability (via the neutral red test) were assessed, and the expression of MT-2 and HSP72 were quantified. Not only has the study revealed that *A. chlorotica* is a suitable model for studies on heavy metal impact on immunity, but it showed differential modes of the response. In fact, the impact on body malformation and earthworm mortality was weakest in response to potentially most dangerous (nonessential) cadmium and the strongest mortality towards copper, which in low doses is essential for normal functioning.

## Materials and Methods

### Animals and Exposure Condition

Adult (clitellate) *Allolobophora chlototica* (Sav.) were field collected (unpolluted experimental garden at the Institute of Zoology, Jagiellonian University, Krakow, Poland) and maintained in controlled laboratory conditions (16 ± 1 °C; 12:12 LD) in unpolluted soil samples for at least 2 weeks for acclimatization. The experiments were conducted according to the standard filter paper contact method (Homa et al. 2005; OECD 1984). Briefly, after 3 h on moist filter paper, each worm (0.31 ± 0.075 g body weight, *n* = 3–10) was washed, dried, and placed individually in 50-ml vials filled with filter paper that was soaked with either tap water (control) or a heavy metal chloride solution (11, 22, 44, 66, 88, or 176 µg/ml, which corresponds to 0.33, 0.66, 1.32, 1.98, 2.64, 5.28 µg/cm^2^ of heavy metals). In addition, control animals were kept in natural soil. Doses of heavy metals were selected based on previous publications (Homa et al. 2005, 2010; Plytycz et al. 2011) and adopted to cover a range of several dilutions not to overlook effects of any dose. The highest doses were chosen based on pilot studies in which their impact on earthworm mortality was monitored. *A. chlorotica* turned out to be very sensitive to Cu as three highest doses (66, 88, and 176 µg/ml) were fatal for the worms. For this reason, we were only able to obtained results when applying up to 44 µg/ml of Cu, and we only show mortality (100 %) for the higher dose (66 µg/ml) as an example.

### Harvesting of Coelomocytes

After 3 days exposure to the heavy metals, earthworms were inspected to identify morphological abnormalities and photographed. Subsequently, earthworms were placed individually in Petri dishes containing phosphate buffered saline (PBS, Gibco) and stimulated for 1 min with a 4.5-V electric current to expel coelomic fluid containing coelomocytes through the dorsal pores, according to the procedure described previously (Homa et al. 2005; Roch 1979). Extruded coelomocytes were used for tests described below.

### Flow Cytometric Measurement and Analyses

To determine the cell composition of the coelomic fluid, the coelomic fluid samples were analysed with a FACScalibur flow cytometer (BD Biosciences). During analytical experiments, 10,000 threshold events per worm sample were collected and analysed for forward scatter (FSC) (for cell size) and sideward scatter (SSC) (cell complexity) properties. Fluorescence FL1-H (emission 530 nm; excitation 488 nm) was recorded to detect autofluorescent eleocytes (Homa et al. 2010).

### Neutral Red Uptake

To determine neutral red (NR) uptake by cells, the extruded coelomocytes were incubated for 10 min in the presence of the NR solution (Sigma-Aldrich; in PBS) at a final concentration of 40 µg/ml as described previously (Weeks and Svendsen 1996). Then, the cells were analysed by means of a FACScalibur flow cytometer (Plytycz et al. 2007). By producing density plots of FL2-H (neutral red-derived fluorescence) versus side scatter SSC (indicating cell complexity/granularity) allowed the proportion of neutral red accumulating cells to the NE^-^ cells to be quantified. The resulting data were analyzed with the WinMDI 2.9 software (Joe Trotter, http://facs.scripps.edu).

### Immunohistochemistry Detection of Metallothionein (MT-2)

Coelomocytes from individuals earthworms were used for cytospin preparations as described previously (Homa et al. 2005). For cross-section preparations, segments posterior to the clitellum were fixed for 4 h in 2 % paraformaldehyde dissolved in PBS and then washed for 1 h in 12 % saccharose, and overnight in 25 % saccharose (both dissolved in PBS) (Baumann 1997). Subsequently, tissues were frozen in liquid nitrogen and sectioned (7 µm) on a cryostat (Shandon OT, Astmoor, Runcorn, Cheshire, UK), fixed with solution of 70 % cold ethanol, and stored as frozen samples (−20 °C) until further analyses. Next, the cross-section preparations were subjected to immunoperoxidase staining with rabbit polyclonal antibody raised against metallothionein 2 (wMT-2) from *Lumbricus rubellus* (Hoeckner et al. 2015; Morgan et al. 2004; Stürzenbaum et al. 2001) diluted 1:500 and a secondary goat anti-rabbit IgG_1_ antibody conjugated with horseradish peroxidase (Sigma-Aldrich Co., St. Louis, MO; dilution 1:1000). The reaction was developed using DAB (3,3′-diaminobenzidine) HRP substrate (Sigma-Aldrich) and counter stained with hematoxylin for cell nuclei visualization (Stamar, Poland). The sections were imaged and photographed with a Jenamed-2 microscope (Carl Zeiss Jena) fitted with Nikon digital camera (Coolpix 4500).

### Immuno-Blot Detection of Heat Shock Protein HSP72 and MT-2

To examine expression of stress proteins, dot-blot assays were performed in a 96-well plate format using a Bio-dot microfiltration manifold (Bio-Rad, Hercules, CA). Lysates of coelomocytes were prepared according to the manufacturer’s protocols (Roche Applied Diagnostic GmbH, Mannheim, Germany) and as described previously by Homa et al. (2005). First, protease inhibitor cocktail PMSF (Roche) was used to prepare coelomocyte extracts. The amount of protein was determined by the BCA method (Sigma Aldrich), and samples were adjusted to an equal concentration. Subsequently, 50 µl of samples (35 µg of protein) was added to each well of the microfiltration apparatus and the blotting was performed according to a standard (Bio-Rad). Then, the membranes were blocked for 45 min at 37 °C in a blocking buffer containing 5 % non-fat milk solution (Gostyń) dissolved in TBS (20 mM Tris–HCl, 500 mM NaCl, pH 7.5). Next, the membranes were treated with monoclonal anti-HSP72 biotin conjugated antibody (Stressgen, San Diego, CA) diluted 1:4000 or rabbit polyclonal antibodies raised against MT-2 from *Lumbricus rubellus* (1:1000 in TTBS (TBS with 0.05 % Tween 20) (Homa et al. 2005; Morgan et al. 2004) containing 1 % non-fat milk, and incubated overnight at 4 °C. For MT-2 staining, horseradish peroxidase conjugated secondary goat anti-rabbit IgG_1_antibody (Sigma-Aldrich) diluted 1:10,000 was used. Next, the membranes were washed at room temperature by continuous shaking in TTBS. The HSP72 was immunodetected with Streptavidin–Alkaline Phosphatase (SAv-AKP) (BD Pharmingen, San Diego, CA) after 30 min incubation at room temperature. Then, the reaction was developed by addition of BCIP/NBT substrates (Bio-Rad). The MT-2 reaction was developed using DAB (3,3′-diaminobenzidine) HRP substrate (Sigma-Aldrich). The membranes were air-dried and a densitometric analysis of protein dots was performed using of the UVISoft-UVIMap program (UVItec, Ltd.).

### Data Analysis and Statistics

Results are expressed as means ± standard errors (SE). Significant differences between means were evaluated using one-way ANOVA, and a post hoc Tukey (Statistica, StatSoft) test with the level of significance was established at *p* < 0.05.

## Results

### Viability, Morphological Abnormality, Composition and Number of Coelomocytes

**Fig. 1:**
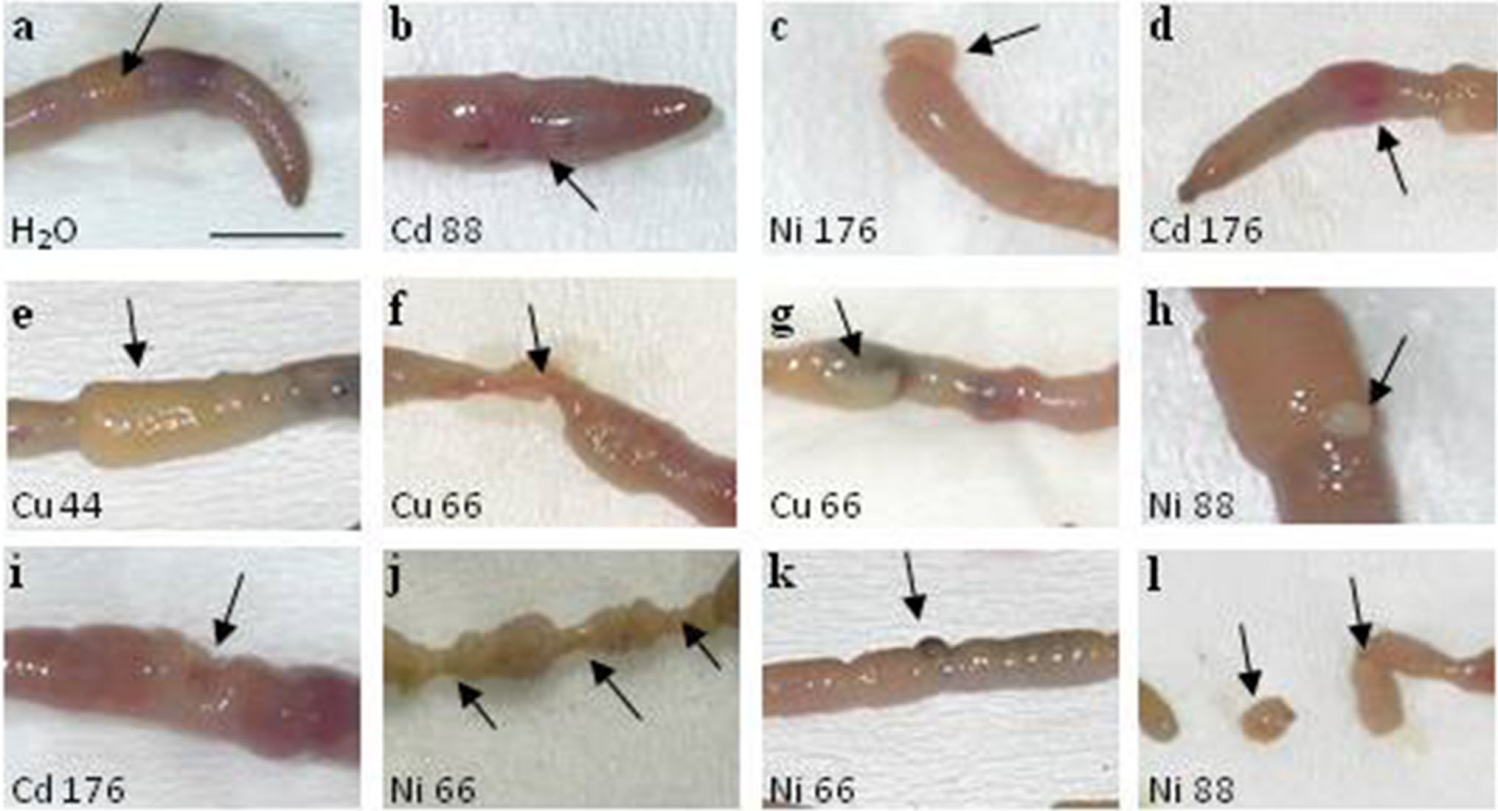
Effect of heavy metals (Ni, Cu, and Cd) on the earthworm *Allolobophora chlorotica* morphology following 3-day exposure on filter paper. Examples of abnormal morphology of the earthworms exposed to different concentrations of the heavy metals (66–176 µg/ml). Control worms were maintained in H_2_O-soaked filter paper. Note intact morphology with visible clitellum (**a**) *scale bar* 1 cm. Exposing earthworms to filter paper soaked with a heavy metal solution resulted in different malformations and swelling of the anterior part (**b**), abnormal shape of the posterior end (**c**), bloody lesions (**d**), abnormal swelling at clitella region (**e**). Abnormalities typically included blistering of the body wall (**f–i**), disruption of metameric segmentation (**j**), rupture in body wall (**k**), and body fragmentation at the posterior region (**l**). *Letters* and *numbers* in each panel denote the metal and corresponding concentration (in µg/ml)

Following a 3-day exposure to heavy metals, significant mortality was observed in earthworms exposed already to 44 µg/ml for Cu (100 % mortality) and 88 µg/ml for Ni (~18 %; Table 1). In contrast, first fatalities due to Cd exposure were detected only at the highest concentration tested (176 µg/ml). Distinct morphological changes were detected in animals exposed to heavy metals (Fig. 1), which were in particular profound in earthworms exposed to following doses of Cu (44 µg/ml) and Ni (88 µg/ml) and the highest dose of Cd (176 µg/ml; Fig. 1). The abnormalities typically included the blistering of the body wall (Figs. 1e–i), bloody lesions (Fig. 1d), vesicles (Fig. 1h), disruption of metameric segmentation (Fig. 1j), abnormal swelling at clitellar region (Fig. 1e), and a body fragmentation at the posterior region (Fig. 1l).

**Table 1:** *Allolobophora chlorotica* mortality and coelomocyte composition in response to heavy metals

	µg/ml	Mortality (%)	C (×10^6^)	A (×10^6^)	E (×10^6^)
Soil	0	0	4.62 ± 0.39^a^	1.99 ± 0.25^a^	2.63 ± 0.30^a^
H_2_O	0	0	4.65 ± 0.41^a^	2.19 ± 0.35^a^	2.46 ± 0.25^a^
Ni	22	0	1.20 ± 0.55^b^	0.88 ± 0.61^a^	0.32 ± 0.12^b^
	44	5.9	2.68 ± 0.48^b^	1.12 ± 0.17^a^	1.56 ± 0.39^b^
	66	12.5	1.08 ± 0.31^b^	0.79 ± 0.33^ab^	0.28 ± 0.07^b^
	88	17.6	1.93 ± 0.64^b^	1.10 ± 0.35^a^	0.82 ± 0.29^b^
	176	66.7	nt	nt	nt
Cu	11	0	1.64 ± 0.50^b^	0.79 ± 0.24^b^	0.85 ± 0.28^b^
	22	7.7	2.28 ± 0.44^b^	1.26 ± 0.23^ab^	1.02 ± 0.24^b^
	44	83.3	1.04 (NS)	0.50 (NS)	0.54 (NS)
	66	100	nt	nt	nt
Cd	22	0	3.98 ± 0.64^a^	2.63 ± 0.53^a^	1.35 ± 0.21^ab^
	44	0	4.03 ± 0.56^*a*^	2.13 ± 0.35^a^	1.89 ± 0.25^ab^
	66	0	4.30 ± 0.43^a^	2.33 ± 0.48^a^	1.98 ± 0.52^ab^
	88	0	3.54 ± 0.53^a^	1.63 ± 0.22^a^	1.92 ± 0.41^ab^
	176	30	3.30 ± 0.75^a^	1.88 ± 0.69^a^	1.42 ± 0.41^b^

Control earthworms were maintained either in soil or on water (H_2_O)-soaked filter paper. Experimental animals were exposed to different concentrations of Ni, Cu, and Cd for 3 days. Amebocytes (A), eleocytes (E), and total coelomocyte counts (C) were determined using a hemocytometer, mean ± SE, *p* < 0.05

Different letters (a vs. b) indicate that mean values are statistically significantly different from soil controls

*NS* no statistical analyses was performed due to high mortality, *nt* not tested

The number and composition of coelomocytes was very similar in worms maintained in control (unpolluted) soil or exposed for 3 days to H_2_O soaked filter paper. In contrast, exposure to Cu and Ni significantly decreased the number of coelomocytes, especially eleocytes. Although no statistically significant changes were observed in Cd exposed worms, there was a tendency towards altered composition of amebocytes but not eleocytes (Table 1). Altogether, concentrations of the tested heavy metals required to kill half of the individuals in given experimental groups were as follows: LC50Ni = 176, LC50Cu = 44, and LC50Cd = 176 µg/ml.

### Flow Cytometric Measurements and Neutral Red Uptake

**Fig. 2:**
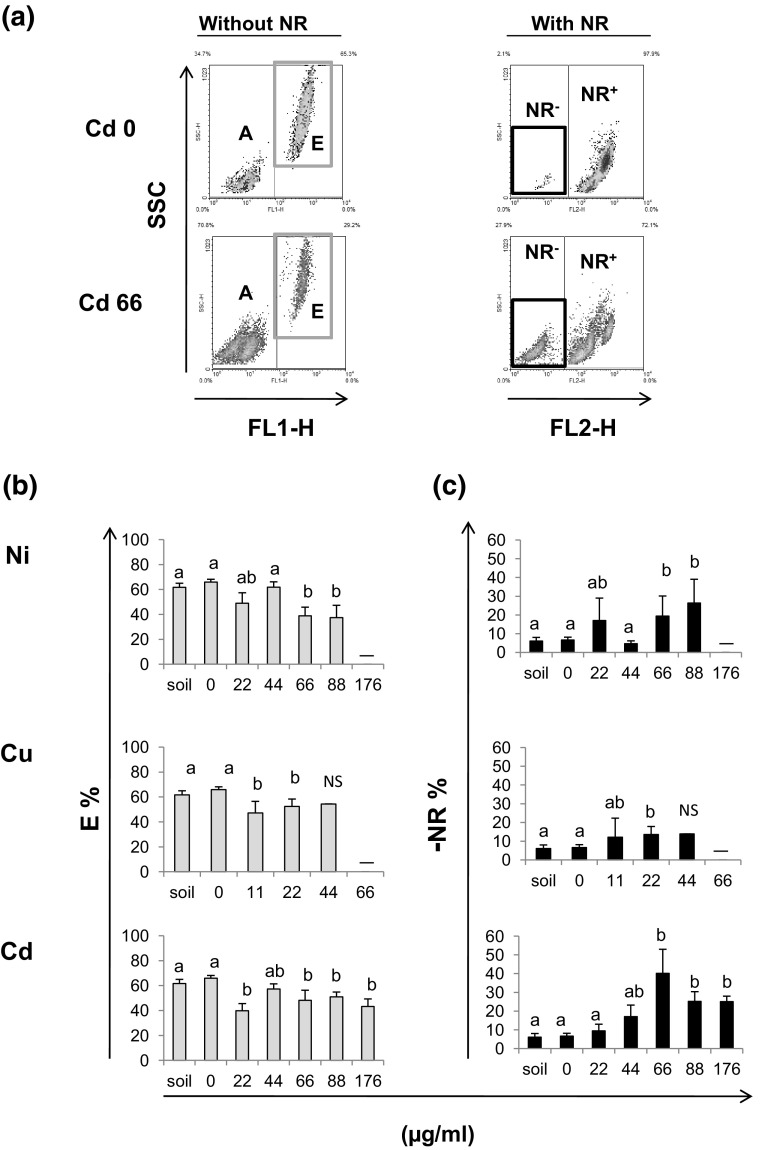
Flow cytometric analysis of coelomocytes derived from earthworms (*Allolobophora chlorotica*) maintained in soil or exposed to filter paper soaked either with H_2_O (control) or heavy metals: Ni, Cu, Cd (11–176 µg/ml). **a** Representative density plots of coelomocytes derived from control worms (Cd 0) and worms exposed to cadmium (Cd 66, 66 µg/ml). Cells were incubated in vitro for 10 min in the presence or absence of Neutral Red dye (NR). Without NR: plots of FL1-H autofluorescence versus cell complexity (SSC-H). Gates set on the density plot indicate two populations of coelomocytes: amoebocytes (A), and eleocytes (E) (left panel). With NR: FL2-H fluorescence versus cell complexity (SSC-H). Gates set on the density plot indicate two populations of coelomocytes: cells that did not absorb NR (NR^−^), and the cell that took in NR (NR^+^). **b** Ratio of autofluorescent eleocytes (E  %) and **c** ratio of coelomocytes that did not absorb NR (NR^−^ %). Mean ± SE, *n* = 3–10, *p* < 0.05. Different letters (*a* vs. *b*) indicate that mean values are statistically significantly different from controls

Compared with unexposed control earthworms, the percentage of eleocytes decreased upon exposure to any of the three metals (Figs. 2a left, b). More than 90 % of cells took up the neutral red dye within the 10-min exposure period (Fig. 2a upper right); however, coelomocytes derived from animals exposed to heavy metals were marked by an impaired uptake of NR (Fig. 2a lower right, representative data). If NR was not added, there was not any signal in FL2-H channel (not shown). Although the ratio of NR-negative (NR^−^) cells was higher in Ni and Cu exposed samples (compared with the control group, data not shown), the difference was less obvious when adjusted to cell numbers (Fig. 2b, c). However, the exposure to Cd significantly decreased the uptake of NR (more NR^−^ cells) either when expressed as ratio of NR^+^/NR^−^ (Fig. 2a lower right) or plotted against coelomocyte numbers (Fig. 2c).

### Immunodetection of MT-2

**Fig. 3:**
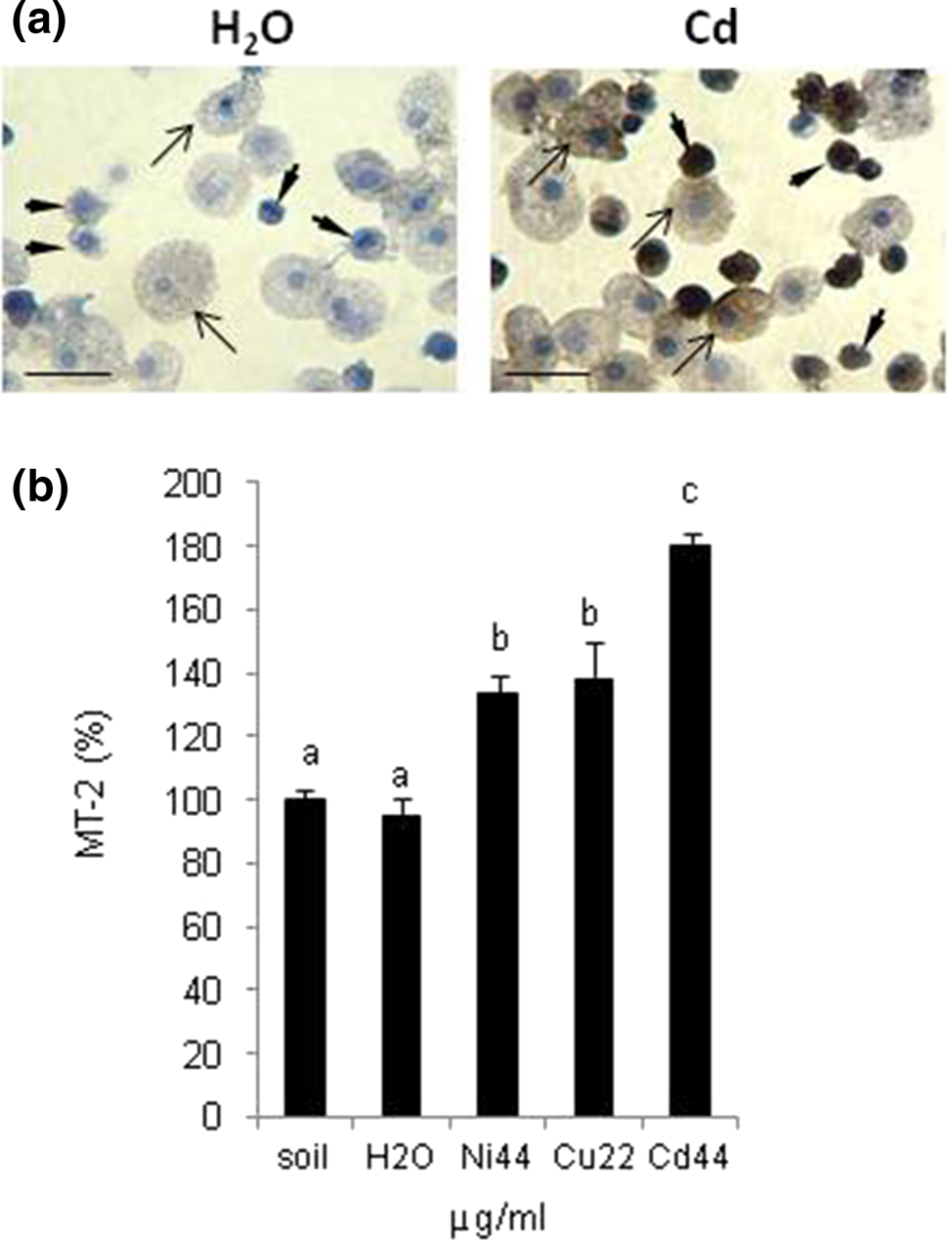
Expression of metallothionein MT-2 in coelomocytes extracted from *Allolobophora chlorotica* exposed to heavy metals. **a** Representative images of immunohistochemical detection of MT-2 (*brown* deposit) in coelomocytes of earthworms maintained on filter paper soaked with H_2_O (control) or exposed to Cd (44 µg/ml) for 3 days. Both amebocytes (*arrowheads*) and eleocytes (*arrows*) were MT-2 positive. *Scale bar* 25 µm. **b** Expression of MT-2 also was evaluated in all cell lysates of coelomocytes derived from animals exposed to Ni, Cu, and Cd at concentrations of 44, 22, and 44 µg/ml, respectively. Mean ± SE, *n* = 3–10, *p* < 0.05. Different letters (*a* vs. *b*) indicate that mean values are statistically significantly different from controls

**Fig. 4:**
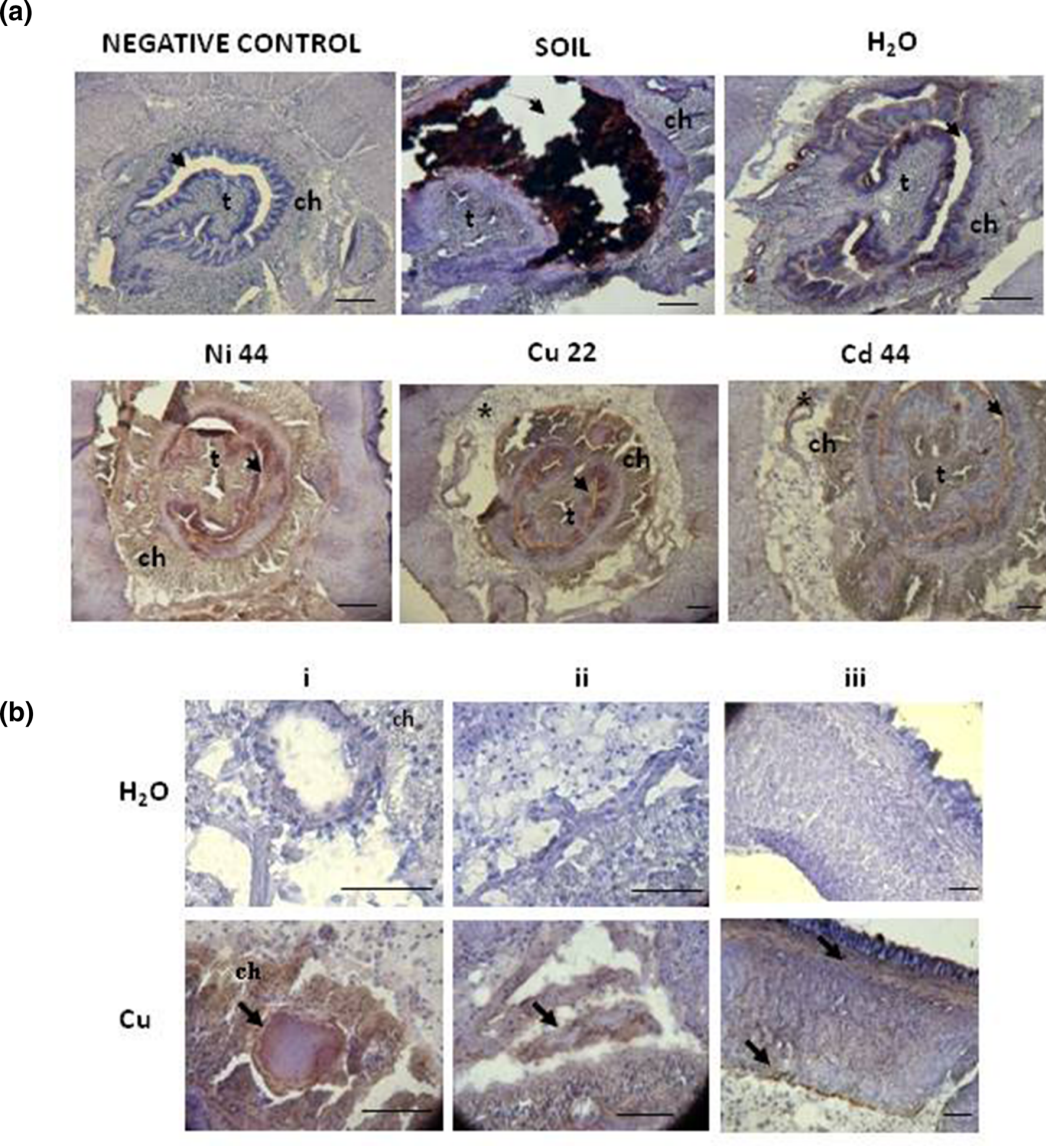
Expression of metallothionein MT-2 in different body compartments of *Allolobophora chlorotica*. Cross-sections of earthworms were obtained from control animals maintained in soil or on filter papers soaked with H_2_O. Experimental groups were maintained on filters soaked with heavy metals for 3 days: Ni, Cu, Cd (11–176 µg/ml). **a** Immunohistochemical evaluation of MT-2 expression. Negative control (lacking the primary antibody) confirmed the absence of false-positive staining; immunoperoxidase (anti-MT-2) stained as brown deposits indicates location of MT-2. *t* typhlosole; *ch* chloragogen cells; *asterisk* free coelomocytes; *arrows* intestinal epithelial cells in the apical regions adjacent to the gut lumen (*arrows*); *scale bar* 100 µm. **b** Representative images showing positive staining for MT-2 after 3-day exposure to Cu (22 µg/ml): arrows indicate; *i* dorsal blood vessel and chloragogen cells (ch), *ii* region of the nephridium, *iii* section through the body wall

Immunohistochemical analyses revealed that the expression of MT-2 was modulated in coelomocytes (Fig. 3a, b) and in body compartments (Fig. 4a, b) of Ni, Cu, and Cd exposed earthworms. While base line levels of MT-2 were detected in coelomocytes derived from control earthworms, the expression increased in the cells extracted from heavy metal exposed worms (Fig. 3a, representative images from the Cd exposed animals). To confirm expression of MT-2 in other cell compartments, cell lysates were prepared and the expression was evaluated by Dot-blotting (Fig. 3b). The signal was significantly elevated in animals exposed to Ni, Cu, and in particular Cd. Interestingly, MT-2 levels also increased in various other tissues and vital organs (Fig. 4a, b), including the chloragogenous tissue, the typhlosolar fold and in intestinal epithelial cells adjacent to the gut lumen (Fig. 4a). Elevated MT-2 expression also was evident in the body wall (the entry point of metals), the nephridia (implicated in the removal of metabolites), and blood vessels (involved with the circulation and distribution of nutrients/contaminants; Fig. 4b). It should be noted at this stage that the weak signal was present in animals exposed to H_2_O via the filter paper test but not in the worms maintained in soil (Fig. 4a).

### Immunoblot-Detection of Heat Shock Proteins HSP72

**Fig. 5:**
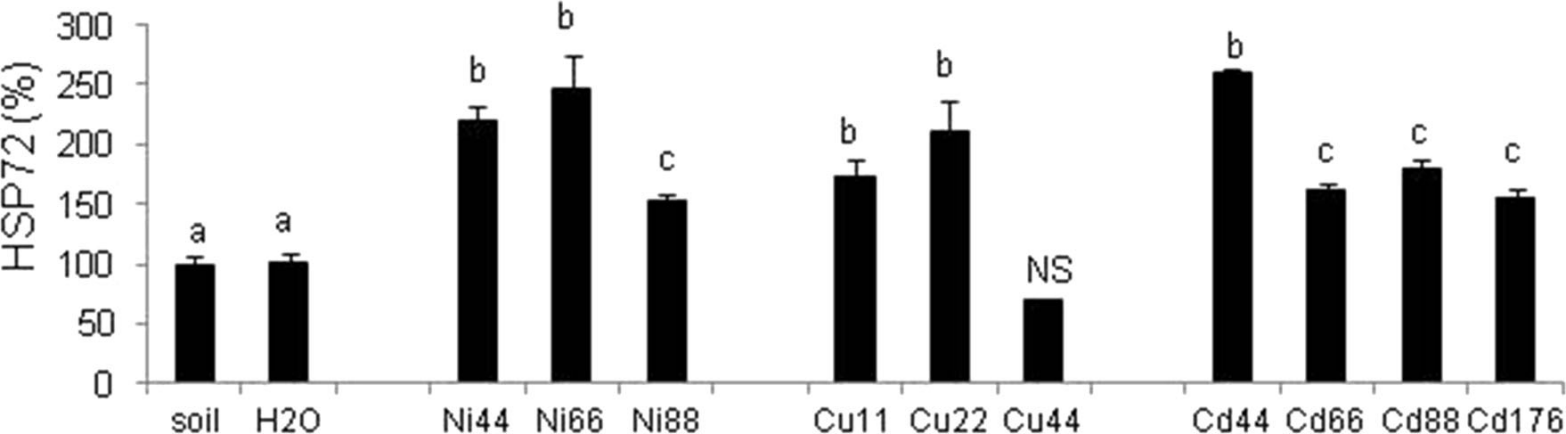
Dot-blotting detection of stress proteins HSP72 in coelomocyte lysates from *Allolobophora chlorotica*. Expression of HSP72 was performed with lysates of cells obtained from animals maintained in soil or exposed for 3 days to filters soaked either with water (H_2_O) or heavy metals, Ni, Cu, and Cd, in concentration of 11–176 µg/ml. Densitometric analysis of protein dots. Mean ± SE, *n* = 3–10, *p* < 0.05. Different letters (*a* vs. *b*) indicate that mean values are statistically significantly different from controls. *NS* no statistical analysis performed due to high mortality

The expression of heat shock proteins HSP72 in coelomocytes lysates was upregulated in animals exposed to any of the three heavy metals. Although high doses of heavy metals significantly induced the expression of HSP72, the most pronounced increase was observed at the lower exposure range (Ni 44/66, Cu 11/22, and Cd 44 µg/ml; Fig. 5). The HSP72 signal in the 44 µg/ml Cu sample was lower than the control group; however, data were derived from a single individual, namely the sole survivor following the 3-day treatment regime (Fig. 5).

## Discussion

Earthworms commonly inhabit soils and litter layers, promote soil biodiversity, soil fertility, and soil health and thus are important indicators of environmental transformations and pollution (Palm et al. 2013). Although earthworm species can populate different niches, they form part of an ecological guild that is not universal to any environment (Paoletti 1999). Indeed, earthworms are classified into three ecological life types: anecics (e.g., *Eisenia fetida*, *Dendrobaena veneta*), endogeics (e.g., *Allolobophora chlorotica*), and epigeics (e.g., *Lumbricus rubellus*). The classification takes into account the structure of their burrows, but most importantly, the three ecological groups differ in their importance in soil functioning (Palm et al. 2013). Accordingly, they might respond to soil contamination in distinct ways, which will influence their utility as bioindicators.

Whilst epigeic and anecic earthworms are routinely studied, few have focused on endogeic species. This study aims to redress this shortfall by evaluating the sensitivity of the endogeic *Allolobophora chlorotica* to heavy metals. In detail, several potential biomarkers of stress/intoxication were measured, including viability and changes in morphology of animals, numbers and activity of coelomocytes, induction of heat shock proteins (HSP72), and metallothioneins (MT-2). The results demonstrate that exposure to copper, nickel, and cadmium chlorides induced significant changes in all of the studied biomarkers in *A. chlorotica*. However, the degree of induced changes differed between the metals, with Ni and Cu inducing highest mortality and most profound morphological changes, and Cd exposure leading to the strongest expression of cytoprotective proteins. No mortality or any symptoms of morphological changes were observed in the control group (H_2_O exposed) throughout the experiments, whereas death and morphological abnormalities were frequently encountered in animals exposed to Ni, Cu, and Cd. This is in line with some previous reports showing that morphological and histological endpoints can be a useful tool to evaluate pesticide and heavy metals toxicity (Venkateswara Rao et al. 2003; Venkateswara Rao and Kavitha 2004). Regarding mortality and morphological changes, our data revealed that *A. chlorotica* exhibits a differential sensitivity towards heavy metals.

Previous studies have reported the effect on *E. fetida* exposed to leaded gasoline (tetra ethyl lead), lead oxide, chlorpyrifos, and organophosphorus insecticide on the filter papers (Chakra Reddy and Venkateswara Rao 2008; Venkateswara Rao et al. 2003; Venkateswara Rao and Kavitha 2004). Pathological symptoms (many of which resembled those observed in our current study) included body swelling, curling, excessive mucus secretion, increase in clitellar region, breakage in body wall, bloody lesion, vesicles, disruption of metameric segmentation, abnormal, and fragmentations at posterior region (Venkateswara Rao et al. 2003; Venkateswara Rao and Kavitha 2004; Chakra Reddy and Venkateswara Rao 2008).

According to Scott-Fordsmand, one of the most sensitive toxicological parameter in the case of *Eisenia veneta* is the lysosomal membrane stability of coelomocytes (Scott-Fordsmand et al. 1998). We used a modified test, utilizing flow cytometry, to measure neutral red (NR) accumulation in earthworm coelomocytes (Plytycz et al. 2007). NR is a vital dye that accumulates in the lysosomes of live cells. We showed that in the case of all tested heavy metals (Cu, Ni, and Cd), a significant number of coelomocytes did not take in (accumulate) NR. An effect that was particularly pronounced in animals exposed to Cd. Similar results were observed using coelomocytes extracted from *Lumbricus terrestris* (Fugère et al. 1996), *E. fetida* and *Dendrobaena veneta* (Homa et al. 2005; Olchawa et al. 2006). In addition, we explored whether protective mechanisms were activated in *A. chlorotica* exposed to heavy metals. In earthworms, similar to other species, exposure to heavy metals can lead to DNA damage (Fourie et al. 2007; Reinecke and Reinecke 2004), apoptosis mediated by caspases (Homa et al. 2007) and perturbations in stability of cell membrane (Asensio et al. 2007; Plytycz et al. 2007; Scott-Fordsmand et al. 1998; Weeks and Svendsen 1996). In response, defense mechanisms are activated, which include the expression of heat shock proteins (Homa et al. 2003, 2005), metal-binding proteins (Homa et al. 2007; Morgan et al. 2004), or glutathione transferases (Farombi et al. 2007). In the current study, the expression of HSP72 was highest at a relatively low dose of the three heavy metals. In agreement with this, coelomocytes derived from those animals exhibited an enhanced accumulation of NR and overall viability. This may suggest that HSPs protect cells from stress-induced, caspase-dependent apoptosis (Gupta et al. 2010; Parcellier et al. 2003); however, HSP72 expression was lower at higher metal concentrations. This finding aligns well with our previous study on *A. chlorotica*, which reported that the reduction in HSP70 and HSP72 detection was correlated to higher levels of cell death mediated by caspase 3 (Homa et al. 2007).

Metal binding metallothioneins (MTs) are important players in the metal detoxification pathway (Dallinger 1996; Morgan et al. 2004). Previous studies have demonstrated that an antibody raised against *Lumbricus rubellus* MT-2 (Stürzenbaum et al. 2001, 2004) is sufficiently cross-reactive to allow the induction of MT-2 protein to be assessed in coelomocytes derived from *E. fetida, D. veneta* and *A. chlorotica* (Homa et al. 2005, 2007, 2010; Olchawa et al. 2006). Likewise, transcript quantification of MT-2 (mRNA) revealed that at least one metallothionein isoform is strongly inducible by Cd in *A. chlorotica* (Homa et al. 2010). The results presented in the current paper confirm these preliminary findings and by application of immunocytochemical and immunoblotting techniques extends the knowledge base to include valuable information surrounding the dose and time resolved expression of MT-2 in coelomocytes and other body compartments. Animals exposed to heavy metals via moist filter paper presented a strong MT-2 signal in the chloragogenous tissue surrounding the gut, the typhlosolar fold as well as the nephridia, blood vessels, and epithelial cells of the intestine. Noteworthy is the fact that MT-2 expression was observed in intestinal epithelial gut cells in worms exposed to filter paper soaked only in H_2_O. Others have shown that starvation can lead to increased expression of MTs (Hashemi et al. 2008; Sogawa et al. 2003), which might explain this finding (given that earthworms were not fed for 3 days during the filter paper exposure). Similar pattern of MT-2 expression were reported to occur in *L. rubellus* exposed to metals in contaminated soil (Hoeckner et al. 2015; Morgan et al. 2004). However, unlike *L. rubellus*, we also detected MT-2 expression in the body wall, which might reflect the differences in exposure conditions or indeed pinpoint species-specific differences in metal handing/distribution. Further experiments would be needed to explore this notion in more detail.

In summary, the exposure of *A. chlorotica* to metals induces strong phenotypic responses (mortality and changes of body morphology) in the order Cd < Ni ≤ Cu; however, the sensitivity towards metals in terms of the expression of key stress proteins/metalloproteins (HSP72 and MT-2) is reversed to Cd > Ni ≥ Cu. However, the degree of induced changes differed between the metals; Ni and Cu induced highest mortality and most profound morphological changes, and Cd exposure led to the strongest expression of cytoprotective proteins. LC50 concentrations of the tested heavy metals were consequently lowest for Cd and only high doses of Ni and Cu killed half of the tested earthworms. These rather unexpected results might be explained by a fact that cadmium is the most poisoning of the tested metals, but in contrast to Ni (present endogenously in unpolluted animals) and Cu (e.g., involved in haemoglobin production), it is not essential for any known biological process. Therefore, Cd triggers much stronger immunobiological response of *A. chlorotica* than Ni or Cu, which might be naturally present not only in soil but also in the earthworm body. Overall, our study reveals that endogeic earthworms represent an interesting model of ecotoxicological studies and shows that *A. chlorotica* is better adopted to protect itself against toxic elements that are not essential for its existence.

## References

[CR1] Asensio V, Kille P, Morgan AJ, Soto M, Marigomez I (2007). Metallothionein expression and neutral red uptake as biomarkers of metal exposure and effect in *Eisenia fetida* and *Lumbricus terrestris* exposed to Cd. Eur J Soil Biol.

[CR2] Baumann O (1997). Biogenesis of surface domains in fly photoreceptor cells: fine-structural analysis of the plasma membrane and immunolocalization of Na^+^, K^+^ ATPase and α-spectrin during cell differentiation. J Comp Neurol.

[CR3] Calisi A, Lionetto MG, Schettino T (2009). Pollutant-induced alterations of granulocyte morphology in the earthworm *Eisenia foetida*. Ecotoxicol Environ Saf.

[CR4] Calisi A, Lionetto MG, De Lorenzis E, Leomanni A, Schettino T (2014). Metallothionein induction in the coelomic fluid of the earthworm *Lumbricus terrestris* following heavy metal exposure: a short report. Biomed Res Int.

[CR5] Chakra Reddy N, Venkateswara Rao J (2008). Biological response of earthworm, *Eisenia foetida* (Savigny) to an organophosphorous pesticide, profenolos. Ecotoxicol Environ Saf.

[CR6] Cooper EL, Stein EA, Ratcliffe NA, Rowley AF (1981). Oligochaetes. Invertebrate blood cells.

[CR7] Cossarizza A, Cooper EL, Suzuki MW, Salvioli S, Capri M, Gri G, Quaglino D, Franceschi C (1996). Earthworm leukocytes that are not phagocytic and cross-react with several human epitopes can kill human tumor cell lines. Exp Cell Res.

[CR8] Dallinger R (1996). Metallothionein research in terrestrial invertebrates: synopsis and perspectives. Comp Biochem Physiol C.

[CR9] Engelmann P, Kiss J, Csöngei V, Cooper EL, Németh P (2004). Earthworm leukocytes kill HeLa, HEp-2, PC-12 and PA317 cells in vitro. J Biochem Biophys Methods.

[CR10] Farombi EO, Adelowo OA, Ajimoko YR (2007). Biomarkers of oxidative stress and heavy metal levels as indicators of environmental pollution in African catfish (*Clarias gariepinus*) from Nigeria Ogun River. Int J Environ Res Public Health.

[CR11] Field SG, Kurtz J, Cooper EL, Michiels NK (2004). Evaluation of an innate immune reaction to parasites in the earthworms. J Invertebr Pathol.

[CR12] Fisher E, Molnár L (1992). Environmental aspects of the chloragogenous tissue of earthworms. Soil Biol Biochem.

[CR13] Fourie F, Reinecke SA, Reinecke AJ (2007). The determination of earthworm species sensitivity differences to cadmium genotoxicity using the comet assay. Ecotoxicol Environ Saf.

[CR14] Fugère N, Brousseau P, Krzystyniak K, Coderre D, Fournier M (1996). Heavy metal-specific inhibition of phagocytosis and different in vitro sensitivity of heterogeneous coelomocytes from *Lumbricus terrestris* (Oligochaeta). Toxicology.

[CR15] Giska I, van Gestel CA, Skip B, Laskowski R (2014). Toxicokinetics of metals in the earthworm *Lumbricus rubellus* exposed to natural polluted soils: relevance of laboratory tests to the field situation. Environ Pollut.

[CR16] Gupta SC, Sharma A, Mishra M, Mishra RK, Chowdhuri DK (2010). Heat shock proteins in toxicology: how close and how far?. Life Sci.

[CR17] Hashemi S, Kunwar PS, Blust R, De Boeck G (2008). Differential metallothionein induction patterns in fed and starved carp (*Cyprinus carpio*) during waterborne copper exposure. Environ Toxicol Chem.

[CR18] Hoeckner M, Dallinger R, Stürzenbaum SR (2015). Metallothionein gene activation in the earthworm (*Lumbricus rubellus*). Biochem Biophys Res Commun.

[CR19] Homa J, Niklińska M, Plytycz B (2003). Effect of heavy metals on coelomocytes of earthworm *Allolobophora chlorotica*. Pedobiol.

[CR20] Homa J, Olchawa E, Stürzenbaum SR, Morgan AJ, Plytycz B (2005). Early phase immunodetection of metallothionein and heath shock proteins in extruded earthworm coelomocytes after dermal exposure to metal ions. Environ Pollut.

[CR21] Homa J, Stürzenbaum SR, Morgan AJ, Plytycz B (2007). Disrupted homeostasis in coelomocytes of *Eisenia fetida* and *Allolobophora chlorotica* exposed dermally to heavy metals. Eur J Soil Biol.

[CR22] Homa J, Klimek M, Kruk J, Cocquerelle C, Vandenbulcke F, Plytycz B (2010). Metal-specific effects on metallothionein gene induction and riboflavin content in coelomocytes of *Allolobophora chlorotica*. Ecotoxicol Environ Saf.

[CR23] Honeycutt ME, Roberts BL, Roane DS (1995). Cadmium disposition in the earthworm *Eisenia fetida*. Ecotoxicol Environ Saf.

[CR24] Kalaç Y, Kimiran A, Ulakoğlu G, Çotuk A (2002). The role of opsonin in phagocytosis by coelomocytes of the earthworm *Dendrobaena venata*. J Cell Mol Biol.

[CR25] Kammenga JE, Dallinger R, Donker MH, Köhler HR, Simonsen V, Triebskorn R, Weeks JM (2000). Biomarkers in terrestrial invertebrates for ecotoxicological soil risk assessment. Rev Environ Contam Toxicol.

[CR26] Kiang JG, Tsokos GC (1998). Heat shock protein 70 kDa: molecular biology, biochemistry, and physiology. Pharmacol Ther.

[CR27] Kooch Y, Jalilvand H (2008). Earthworms as ecosystem engineers and the most important detritivors in forest soils. Pak J Biol Sci.

[CR28] Kurek A, Homa J, Kauschke E, Plytycz B (2007). Characteristics of coelomocytes of the stubby earthworm, *Allolobophora chlorotica* (Sav.). Eur J Soil Biol.

[CR29] Leitão S, Moreira-Santos M, Van den Brink PJ, Ribeiro R, José Cerejeira M, Sousa JP (2014). Ethoprophos fate on soil-water interface and effects on non-target terrestrial and aquatic biota under Mediterranean crop-based scenarios. Ecotoxicol Environ Saf.

[CR30] Liess M (2002). Population response to toxicants is altered by intraspecific interaction. Environ Toxicol Chem.

[CR31] Luan W, Li F, Zhang J, Wen R, Li Y, Xiang J (2010). Identification of a novel inducible cytosolic Hsp70 gene in Chinese shrimp *Fenneropenaeus chinensis* and comparison of its expression with the cognate Hsc70 under different stresses. Cell Stress Chaperones.

[CR32] Maboeta MS, Reinecke SA, Reinecke AJ (2004). The relationship between lysosomal biomarker and organismal responses in an acute toxicity test with *Eisenia fetida* (Oligochaeta) exposed to the fungicide copper oxychloride. Environ Res.

[CR33] Morgan AJ, Stürzenbaum SR, Winters C, Grime GW, Aziz NA, Kille P (2004). Differential metallothionein expression in earthworm (*Lumbricus rubellus*) tissues. Ecotoxicol Environ Saf.

[CR34] Moseley P (2000). Stress proteins and the immune response. Immunopharmacology.

[CR35] Nadeau D, Corneau S, Plante I, Morrow G, Tanguay RM (2001). Evaluation for Hsp70 as a biomarker of effect of pollutants on the earthworm *Lumbricus terrestris*. Cell Stress Chaperones.

[CR36] OECD (1984). Guidelines for the testing of chemicals no. 207 earthworm acute toxicity tests.

[CR37] Olchawa E, Bzowska M, Stürzenbaum SR, Morgan AJ, Plytycz B (2006). Heavy metals effect the coelomocyte-bacteria balance in earthworms: environmental interactions between abiotic and biotic stressors. Environ Pollut.

[CR38] Palm J, van Schaik NLMB, Schröder B (2013). Modelling distribution patterns of anecic, epigeic and endogeic earthworms at catchment-scale in agro-ecosystems. Pedobiologia.

[CR39] Paoletti MG (1999). The role of earthworms for assessment of sustainability and as bioindicators. Agric Ecosyst Environ.

[CR40] Parcellier A, Gurbuxani S, Schmitt E, Solary E, Garrido C (2003). Heat shock proteins, cellular chaperones that modulate mitochondrial cell death pathways. Biochem Biophys Res Commun.

[CR41] Piano A, Valbonesi P, Fabbri E (2004). Expression of cytoprotective proteins, heat shock protein 70 and metallothioneins, in tissues of *Ostrea edulis* exposed to heat and heavy metals. Cell Stress Chaperones.

[CR100] Plytycz B, Klimek M, Homa J, Mazur AI, Kruk J, Morgan AJ (2011). Species-specific sensitivity of earthworm coelomocytes to dermal metal (Cd, Cu, Ni, Pb, Zn) exposures: methodological approach. Pedobiologia.

[CR42] Plytycz B, Klimek M, Homa J, Tylko G, Kolaczkowska E (2007). Flow cytometric measurement of neutral red accumulation in earthworm coelomocytes: novel assay for studies on heavy metal exposure. Eur J Soil Biol.

[CR43] Prentø P (1979). Metals and phosphate in the chloragosomes of *Lumbricus terrestris* and their possible physiological significance. Cell Tissue Res.

[CR44] Reinecke SA, Reinecke AJ (2004). The comet assay as biomarker of heavy metal genotoxicity in earthworms. Arch Environ Contam Toxicol.

[CR45] Roch P (1979). Protein analysis of earthworm coelomic fluid: polymorphic system of the natural hemolysin of *Eisenia fetida andrei*. Dev Comp Immunol.

[CR46] Roesijadi G (1996). Metallothionein and its role in toxic metal regulation. Comp Biochem Physiol.

[CR47] Roots BI, Johnston PV (1966). The lipids and pigments of the chloragosomes of the earthworm *Lumbricus terrestris* L.. Comp Biochem Physiol.

[CR48] Scott-Fordsmand JJ, Weeks JM, Hopkin SP (1998). Toxicity of nickel to the earthworm and the applicability of the Neutral Red Retention Assay. Ecotoxicol.

[CR49] Shackelford RE, Kaufmann WK, Paules RS (1999). Cell cycle control, checkpoint mechanisms, and genotoxic stress. Environ Health Perspect.

[CR50] Sizmur T, Tilston EL, Charnock J, Palumbo-Roe B, Watts MJ, Hodson ME (2011). Impacts of epigeic, anecic and endogeic earthworms on metal and metalloid mobility and availability. J Environ Monit.

[CR51] Sogawa N, Sogawa CA, Fukuoka H, Mukubo Y, Yoneyama T, Okano Y, Furuta H, Onodera K (2003). The changes of hepatic metallothionein synthesis and the hepatic damage induced by starvation in mice. Methods Find Exp Clin Pharmacol.

[CR52] Stürzenbaum SR, Winters C, Galay M, Morgan AJ, Kille P (2001). Metal ion trafficking in earthworms. Identification of a cadmium-specific metallothionein. J Biol Chem.

[CR53] Valembois P, Lassègues M, Roch P (1992). Formation of brown bodies in the coelomic cavity of the earthworm *Eisenia fetida andrei* and attendant changes in shape and adhesive capacity of constitutive cells. Dev Comp Immunol.

[CR54] Valembois P, Seymour J, Lassègues M (1994). Evidence of lipofuscin and melanin in the brown body of the earthworm *Eisenia fetida andrei*. Cell Tissue Res.

[CR55] Venkateswara Rao J, Kavitha P (2004). Toxicity of azodrin on the morphology and acetylcholinesterase activity of the earthworm *Eisenia foetida*. Environ Res.

[CR56] Venkateswara Rao J, Surya Pavan Y, Madhavendra SS (2003). Toxic effects of chlorpyrifos on morphology and acetylcholinesterase activity in the earthworm, *Eisenia foetida*. Ecotoxicol Environ Saf.

[CR57] Weeks JM, Svendsen C (1996). Neutral red retention by lysosomes from earthworm (*Lumbricus rubellus*) coelomocytes: a simple biomarker of exposure to soil copper. Environ Toxicol Chem.

[CR58] Wuana RA, Okieimen FE (2011). Heavy metals in contaminated soils: a review of sources, chemistry, risks and best available strategies for remediation. ISRN Ecol.

